# Peripheral Blood Autoantibodies Against to Tumor-Associated Antigen Predict Clinical Outcome to Immune Checkpoint Inhibitor-Based Treatment in Advanced Non-Small Cell Lung Cancer

**DOI:** 10.3389/fonc.2021.625578

**Published:** 2021-03-16

**Authors:** Juan Zhou, Jing Zhao, Qingzhu Jia, Qian Chu, Fei Zhou, Xiangling Chu, Wencheng Zhao, Shengxiang Ren, Caicun Zhou, Chunxia Su

**Affiliations:** ^1^ Department of Oncology, Shanghai Pulmonary Hospital & Thoracic Cancer Institute, Tongji University School of Medicine, Shanghai, China; ^2^ Department of Oncology, Xinqiao Hospital, Third Military Medical University, Chongqing, China; ^3^ Department of Oncology, Tongji Hospital of Tongji Medical College, Huazhong University of Science and Technology, Wuhan, China

**Keywords:** biomarker, autoantibody, tumor-associated antigen, immune checkpoint inhibitor, lung cancer

## Abstract

**Background:**

Peripheral blood biomarkers to immunotherapy have attracted more and more attentions owing to noninvasive nature. This study was designed to identify a panel of tumor associated autoantibodies (TAAbs) in plasma to predict the clinical outcome of ICIs-based treatment in advanced NSCLC patients and correlation between TAAbs and checkpoint inhibitor pneumonitis (CIP) would also be investigated.

**Materials and Methods:**

Baseline plasma was collected from patients with advanced NSCLC before receiving ICIs-based treatment. ELISA was used to detect concentration of autoantibodies. Clinical efficacy was evaluated according to RECIST v1.1.

**Results:**

We have identified a panel of five-TAAbs to predict responses of ICIs-based treatment in a discovery cohort (n = 37), and confirmed its predictive value in a validation cohort (n = 129). In the validation cohort, the positivity of this 5-TAAbs panel was significantly associated with better response (ORR: 44.4% vs. 13.6%, *P* < 0.001) and longer PFS (7.6 vs. 3.3m, *P* < 0.001). This significant association was remained in subgroup of patients treated with combination therapy (ORR: 43.8% vs. 13.7%, *P* = 0.004,PFS: 6.7 vs. 3.7m, *P* = 0 .017). Furthermore, this 5-TAAs panel worked better in patients who received subsequent-line treatment (ORR: 42.4% vs. 7.7%, *P* = 0.001, PFS: 6.2 vs. 3.0m, *P* = 0.004) than those received first-line treatment (ORR: 46.7% vs. 35.7%, *P* = 0.345, PFS: NR vs. 10.48m, *P* = 0.146). In addition, the CIP incidence in patients with 5-TAAbs positive was significantly higher comparing to negative patients (20.4% vs. 5.9%, *P* = 0.015).

**Conclusion:**

Our 5-TAAbs panel is a potential predictive biomarker for responses and toxicities to ICIs-based treatment in patients with advanced NSCLC.

## Introduction

Immune checkpoint inhibitors (ICIs), targeting programmed cell death-1 (PD-1) and its ligand PD-L1, have significantly prolonged overall survival (OS) of patients with advanced non-small cell lung cancer (NSCLC) (1, 2). However, only around 20% of patients responded to ICIs monotherapy. Combining checkpoint inhibitors with other therapeutics like chemotherapy or anti-angiogenesis therapy has improved objective response rate (ORR) to 47.6%~63.5% (3–6). Therefore, exploring efficacy biomarkers for ICIs-based therapy has been being an essential hotspot in current clinical practice. PD-L1 has been recognized as the current standard biomarker for immunotherapy. However, even in a highly selected population (PD-L1 tumor proportion score≥50%), ORR was only 44.8% for monotherapy, while around 60% for combination therapy in the first-line setting (3, 4, 7). Besides, differences in testing platforms, the various cut-off values for different immunotherapy agents, and the heterogenous nature of PD-L1 expression within tumors have all been points of criticism. Tumor mutational burden (TMB) has been highlighted as another important biomarker irrelative to PD-L1. But it remains great controversial as the OS data from CheckMate 227 revealed a statistically nonsignificant benefit of ipilimumab with nivolumab in patients with high TMB (7). Peripheral blood biomarkers have attracted more and more attentions owing to the relative ease and less invasive nature. Studies suggested that the neutrophil-to-lymphocyte ratio, number of HLA-DR monocytes, activity or number of NK cells, lactate dehydrogenase, and so forth in blood were related to clinical outcome of ICIs-based therapy (8–11). However, the predictive value of these biomarkers needs to be further verified and many other parameters in peripheral blood remain to be clarified to better understand antitumor immune response.

Autoantibodies are produced by activated B cells in response to autologous antigens which are generated by altered protein expression and defect in immune tolerance or inflammation. Autoantibodies have been considered as attractive blood biomarkers for predicting efficacy and toxicity of cancer immunotherapy since they play an important role in the maintenance of host homeostasis. Gowen et al. provided the first evidence that pre-treatment serum antibody profiles were associated with severe immune-related adverse events (irAEs) for anti-CTLA-4 or anti-PD-1 treatment in melanoma (12). de Moel et al. found that autoantibodies developed in 19.2% of patients who were autoantibody-negative pretreatment and autoantibody development following ipilimumab treatment predisposed patients to irAEs under subsequent anti–PD-1 therapy, but patients who developed autoantibodies showed a trend for better survival (Hazard ratio (HR) for all-cause death: 0.66; 95% CI, 0.34–1.26) and therapy response (odds ratio, 2.64; 95% CI, 0.85–8.16) (13). Toi et al. reported that the presence of preexisting antibodies was associated with clinical benefit and development of irAEs in patients with NSCLC treated with nivolumab or pembrolizumab monotherapy (14). Notably, all the above studies have focused on the autoantibodies against to self-antigen like anti-thyroid antibody, antinuclear antibodies, rheumatoid factor, etc. Autoantibody production could also be triggered by tumor associated antigens (TAAs) in cancer patients, which are referred as tumor associated autoantibodies (TAAbs) in the present study. The production of TAAbs is believed to reflect greater immunologic reactivity in cancer patients and enhanced immune surveillance for cancer cells. Two recent studies reported that preexisting TAAbs such as antibody against to NY-ESO-1, XAGE1, and SIX2 correlated with clinical responses to anti-PD-1 monotherapy in NSCLC (15, 16). However, the clinical utility of TAAbs for monitoring efficacy and toxicity to ICIs-based treatment especially combination therapy in lung cancer is less conclusive. In the present study, we aimed to identify a panel of TAAbs to predict the clinical outcome of ICIs-based treatment in advanced NSCLC patients and correlation between TAAbs and irAE occurrence would also be investigated.

## Materials and Methods

### Patients

Patients who have advanced NSCLC with metastatic/recurrent or unresectable stages were enrolled into this study from three medical centers (Shanghai Pulmonary Hospital, Xinqiao Hospital, and Tongji Hospital of Tongji Medical College) between May 2018 and November 2019. Inclusion criteria are as follows: 1) confirmed NSCLC by pathology; 2) staged IIIB/IV according to the eighth edition of the TNM classification for lung cancer; 3) ECOG performance status 0–2. 3) measurable lesions according to Response Evaluation Criteria in Solid Tumors version 1.1 (RECIST v1.1); and 4) received PD-1 inhibitor-based treatment. Exclusion criteria included: 1) autoimmune diseases; 2) received other immunotherapy including but not limiting vaccines and adoptive cellular immunotherapy; 3) active multiple primary malignancies; and 4) receiving intensive immunosuppressive agents.

### Specimen Characteristics

We collected 10 ml peripheral blood of each patient before initiation of ICIs-based treatment within a week, and then centrifuged it to obtain plasma. Plasma was stored at −80 centigrade degree before detection.

### Assay Methods

We used enzyme-linked immunosorbent assay (ELISA) to determine the reactivity of TAAbs. Briefly, TAAs were expressed in *E. coli* and purified *via* multiple steps including affinity chromatography and size exclusion chromatography. The immuno maxisorp 96-well plate (Thermo scientific, #456537) was pre-immobilized with 50 ul of 10 ug/ml of bovine serum albumin (BSA)-biotin (Thermo scientific, #29130). Each TAA protein used in this study contain both streptavidin and Myc tag for purification and quantification purpose. Then, 50 ul of each antigen at a concentration of 150nM was added into microwells and incubated for 1.5 h before assaying for autoantibody level in serum or plasma. A negative control antigen that contained both streptavidin and Myc tag was immobilized in a separate microwell as the background signal. Plasma samples were diluted with phosphate-buffered saline [1:109] and added to the microwells (50 ml/well) for binding of the TAAbs to their respective TAAs. After washing off the extra proteins with washing buffer, horseradish peroxidase -conjugated anti-human IgG was added to each well to bind TAAbs. ELISA substrate 3,3’,5,5’- tetramethylbenzidine was added for color development followed by the addition of stopping solution (1N HCl), and the absorbance was read at optical density (OD) 450 nm on a spectrometer. All incubations were carried out with shaking at room temperature and plates were washed three times with PBS containing Tween 20 (0.1% v/v; Sigma, Poole, UK) between each step. The autoantibody levels by OD measurements were compared to the cutoff OD value determined using healthy control subjects. The cutoff OD value for positive result was calculated as the average plus two times of standard deviation of OD values in healthy control subjects (data did not show). The reliability of this method was confirmed in our previous study (17). A minimal of 95% of specificity in control subjects was observed for five antigens in this study.

### Study Design

This study was retrospectively designed and performed to identify a panel of TAAbs to predict clinical responses and patient survival with ICIs-based treatment, according to the Reporting Recommendations for Tumor Marker Prognostic Studies’ criteria as listed in the guideline.

ICIs-based treatment with monotherapy or combination therapy was administered according to the decision of physicians. When ORR was 30% overall and at least 60% in the TAAbs-positive patients (10, 11), and when the TAAbs-positive proportion was 50% which was indicated in the discovery cohort, the required sample size was 24 in the independent validation set. It was calculated in *a priori* power analysis for Fisher’s exact test with the power level of 0.8 and the significance level of 0.05 by G*Power calculator. Recapitulatory, the whole study was divided into two independent parts, a discovery cohort including 37 patients who received ICIs-based treatment at first-line and a validation cohort including 129 patients treated with ICIs-based treatment at any-line.

The primary endpoint in this study was ORR and progression-free survival (PFS) to ICIs-based treatment, and the secondary endpoint was the incidence of irAEs. To reduce bias of retrospective data, only checkpoint inhibitor pneumonitis (CIP) was selected to be further investigated since it’s occurrence could be traced through computerized tomography images. Efficacy was evaluated according to RECIST v1.1. ORR was complete response plus partial response (PR). Disease control rate (DCR) was complete response plus partial response plus stable disease (SD). PFS was defined as the interval from the initiation of ICIs-based treatment to confirmed disease progression or death of any cause. If disease progression did not occur before the analysis’ deadline or last follow up date, the data would be censored. CIP was diagnosed by experienced oncologists and confirmed by three experienced radiologists independently. The date of CIP diagnosis and highest ICI-pneumonitis grade [according to the fourth Common Toxicity Criteria for Adverse Events (CTCAE) classification] were recorded. If CIP did not occur, the end-time would be censored at last follow up date.

The institutional review board of Shanghai pulmonary hospital approved the study and informed consent of patients were obtained.

### Statistical Analysis

Statistical analyses were performed using SPSS version 22.0. Intergroup comparisons were performed using the Mann–Whitney U test for continuous variables, and Pearson’s χ2 or Fisher’s exact tests for categorical variables. PFS and OS were estimated by Kaplan–Meier method and compared by log-rank test in univariate analyses. Factors with *P*-value < 0.1 in univariate analysis were included to multivariate analysis by the Cox proportional hazards model, which was also used to calculate the HR and corresponding 95% CI. Two-sided *P*-value < 0.05 was considered significant.

## Results

### Characteristics of Study Population

A total of 166 patients with advanced NSCLC who received ICIs-based treatment were enrolled into this study, including a discovery cohort (n = 37) and a validation cohort (n = 129). Baseline characteristics were summarized in **Table 1**. The TAAbs positivity rate in two groups were 51.4% and 48.8%, respectively. In the discovery cohort, patients with squamous lung cancer were inclined to be positive for TAAbs (90% vs. 37%, *P* = 0.008), which was consistant to previously reported data (10, 13). In the validation cohort, patients with only intrathoracic metastases had a lower positivity rate comparing to those with extrathoracic metastases (35.6% vs. 66.1%, *P* = 0.001). Thirty-four patients with EGFR mutation were also included, 2 (4.5%) into the discovery cohort and 32 (25.0%) into the validation cohort. Positivity rates for TAAbs were similar among patients with EGFR mutation, other gene alteration or wild type (37.5% vs. 61.1% vs. 50.6%, *P* = 0.254). In addition, Patients receiving first-line ICIs-based treatment had a higher TAAbs positivity rate than those receiving subsequent-line treatment (68.2% vs. 38.8%, *P* = 0.002). Other clinical factors, such as median age, gender, smoking history, stage, PD-L1 expression level, and treatment regimen were not observed to be significantly correlated with TAAbs positivity.

**Table 1:** Baseline characteristics of patients.

Characteristics	Discovery cohort (n = 37)				Validation cohort (n = 129)			
	all	positive	negative	*P*-value	all	positive	negative	*P*-value
**Age,yr**								
Median, range	63(26-79)	64(49-79)	61(26-73)	0.408	62(30-82)	62.5(36-82)	62(30-76)	0.938
**Gender**								
male	32(86.5)	16(84.2)	16(88.9)	1.000	93(72.1)	48(76.2)	45(68.2)	0.333
female	5(13.5)	3(15.8)	2(11.1)		36(27.9)	15(23.8)	21(31.8)	
**Smoking^#1^**								
no/light	27(73.0)	14(73.7)	13(72.2)	1.000	84(65.1)	37(58.7)	47(71.2)	0.145
heavy	10(27.0)	5(26.3)	5(27.8)		45(34.9)	26(41.3)	19(28.8)	
**ECOG**								
0-1	36(97.3)	19(100.0)	17(94.4)	0.486	123(95.3)	59(93.7)	64(97.0)	0.433
2	1(2.7)	0	1(5.6)		6(4.7)	4(6.3)	2(3.0)	
**Histology**								
non-squa	27(73.0)	10(52.6)	17(94.4)	0.008	96(74.4)	44(69.8)	52(78.8)	0.244
squa	10(27.0)	9(47.4)	1(5.6)		33(25.6)	19(30.2)	14(21.2)	
**Stage**								
IIIB-IIIC	8(21.6)	6(31.6)	2(11.1)	0.232	14(10.9)	7(11.1)	7(10.6)	0.927
IV	29(78.4)	13(68.4)	16(88.9)		115(89.1)	56(88.9)	59(89.4)	
**Metastatic site^#2^**								
intrathoracic	21(56.8)	9(47.4)	12(66.7)	0.236	73(56.6)	26(41.3)	47(71.2)	0.001
bone	10(27.0)	6(31.6)	4(22.2)	0.714	29(22.5)	16(25.4)	13(19.7)	0.438
brain	3(8.1)	1(5.3)	2(11.1)	0.604	27(20.9)	15(23.8)	12(18.2)	0.432
liver	1(2.7)	1(5.3)	0	1.000	12(9.3)	5(7.9)	7(10.6)	0.602
others	5(13.5)	2(10.5)	3(16.7)	0.660	37(28.7)	20(31.7)	17(25.8)	0.452
**PD-L1**								
≥50%	6(30.0)	2(22.3)	4(36.3)	0.850	15(26.8)	9(28.1)	6(25.0)	1.000
1-49%	5(25.0)	3(33.3)	2(18.2)		15(26.8)	8(25.0)	7(29.2)	
negative	9(45.0)	4(44.4)	5(45.5)		26(46.4)	15(46.9)	11(45.8)	
unknown	17	10	7		73	31	42	
**Gene type**								
EGFR mutation	2(5.4)	1(5.3)	1(5.6)	0.230	32(24.8)	12(19.0)	20(30.3)	0.254
other alteration	3(8.1)	3(15.8)	0		18(14.0)	11(17.5)	7(10.6)	
Wild type	32(86.5)	15(78.9)	17(94.4)		79(61.2)	40(63.5)	39(59.1)	
**ICIs Line**								
1	37(100.0)	19(100.0)	18(100.0)	/	44(34.1)	30(47.6)	14(21.2)	0.002
≥2	0	0	0		85(65.9)	33(52.4)	52(78.8)	
**Treatment**								
mono	6(16.2)	2(10.5)	4(22.2)	0.405	30(23.3)	15(23.8)	15(22.7)	1.000
combination	31(83.8)	17(89.5)	14(77.8)		99(76.7)	48(76.2)	51(77.3)	
**ICI agent**								
Nivolumab	4(10.8)	3(15.8)	1(5.6)	0.649	13(10.1)	6(9.5)	7(10.6)	0.798
Pembrolizumab	19(51.4)	10(52.6)	9(50.0)		33(25.6)	18(28.6)	15(22.7)	
Others**^#3^**	14(37.8)	6(31.6)	8(44.4)		83(64.3)	39(61.9)	44(66.7)	

The number (outside the parentheses) and percentage (in the parentheses) of patients in each subgroup were listed in this table. **#1:** unit: package*year. **#2**: What were listed here were the number and percentage of patients with corresponding distant metastatic site. “Intrathoracic” means metastases tumor were confined in thoracic including contralateral pulmonary or pleura metastasis; “bone/brain/liver/others” means whether patient had metastases in these respective organs. **#3**: other ICI agents mainly referred to Chinese domestic PD-1 inhibitors, including camrelizumab, toripalimab, sintilimab, and tislelizumab. yr, year; squa, squamous; mono, monotherapy.

### Determination of the Reactivity of the 5-Tumor-Associated Autoantibodies in the Discovery Cohort

**Figure 1:**
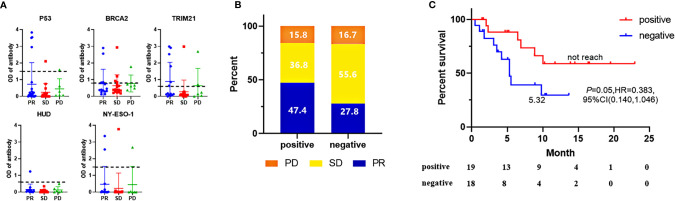
In the discovery cohort, screening results of five “positive correlation” biomarkers **(A)**, A negative value was generated when no corresponding autoantibody signal existed in serum, while the complex matrix effect of serum gave more OD signals in background control well than the well immobilized with a TAAs protein. ORR **(B)** and PFS **(C)** comparison of patients with 5-AABs positive or negative. ORR were compared by fisher’s exact tests and PFS were compared by log-rank test. Two-sided P-value < 0.05 was considered significant.

We first evaluated TAAbs in a set of serum samples from advanced NSCLC patients together with healthy control subjects (data not shown). The 43 TAA proteins, which showed high enough sensitivity (>5%) and specificity (93.6%), were then selected to test in the discovery cohort (**Supplement Table**). These TAAs were categorized based on the correlation of their “positive” score of corresponding autoantibody measurement and clinical response of patients who received ICIs-based treatment. Three categories were classified: 1. “positive correlation”: more than twice patients with PR than PD were positive for these autoantibodies (**Figure 1A**). 2. “negative correlation”: more than twice patients with PD than PR were positive for these autoantibodies. 3. “no correlation”: these did not meet the above two conditions. Antigens in “positive correlation” category included Claudin2, BRCA2, HUD, P53, Annexin1, MAGE-A4, Trim21, TTC14, IMP2, GAGE7, NY-ESO-2, NY-ESO-1; Antigens in “negative correlation” category included ETHE1, AKAP4, PRAME, HSP105, MAGE-A3, KRT8, KRAS, RALA, FEZF1, TTC14, PRAME. Finally, we evaluated the correlation of these 12 antigens from “positive correlation” category with survival in discovery cohort and five TAAbs (p53, BRCA2, HUD, TRIM21, and NY-ESO-1) that performed best were selected. Consistent with published data, the sensitivity of a single autoantibody was low, ranging from 5% to 20%. Therefore, a panel of 5-TAAbs was selected, and the detection result was considered being positive if at least one autoantibody was positive. In contrast, the result was regarded as negative if none of the 5 TAAbs were positive.

As shown in **Figure 1B**, positive results among the antibodies panel in the discovery cohort predicted 47.4% of patients as “PR”, while only 15.8% of patients as “PD”. The sensitivity and specificity of 5-TAAbs for response were 0.643 and 0.565, respectively. The positivity of this 5-TAAbs panel was also associated with better PFS (not reached vs. 5.32 m, P = 0.05, HR = 0.383, 95% CI: 0.140–1.046) (**Figure 1C**).

### Predictive Value of the 5-Tumor-Associated Autoantibody Panel in the Validation Cohort

**Figure 2:**
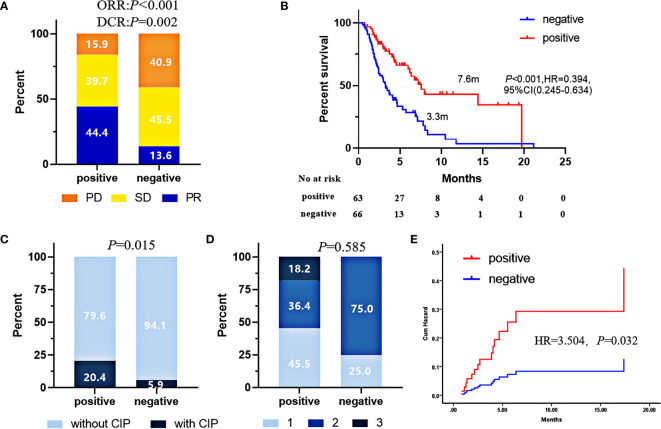
In the validation cohort, ORR **(A)**, PFS **(B)**, CIP incidence **(C)**, CIP grades **(D)**, and risks **(E)** comparison of patients with 5-AABs positive or negative. Pearson’s χ2 test was used to compare ORR, Fisher’s exact tests was used to compare CIP and CIP grades. and PFS were compared by log-rank test. CIP risks were compared by Hazard function of Cox regression. Two-sided P-value < 0.05 was considered significant.

**Figure 3:**
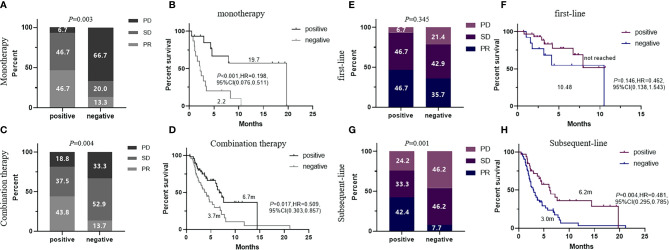
ORR and PFS comparison of patients with 5-AABs positive or negative in subgroup of patients who received ICI monotherapy **(A, B)** and ICI-based combination therapy **(C, D)**, or patients who received immunotherapy at first-line **(E, F)** and subsequent-line **(G, H)**. ORR were compared by fisher’s exact tests and PFS were compared by log-rank test. Two-sided P-value < 0.05 was considered significant.

To confirm the predictive value of the 5-TAAbs panel in heterogeneous patients at clinical setting, we have evaluated how this panel worked in an independent validation cohort which included 129 patients who received ICIs-based treatment at any-line from three hospitals and patients with EGFR mutation were also permitted. In the validation cohort, the positivity of this 5-TAAbs panel was significantly associated with better response (ORR: 44.4% vs. 13.6%, *P* = 0.001; DCR 84.1% vs. 59.1%, *P* = 0.002) and longer PFS (7.6 vs. 3.3 m, *P* < 0.001, HR = 0.394,95% CI: 0.245-0.634) (**Figures 2A, B**). In the subgroup of patients treated with ICIs monotherapy, better response and longer PFS were also observed in TAAbs-positive patients (ORR: 46.7% vs. 13.3%, *P* = 0.003, PFS: 19.7 vs. 2.2 m, *P* < 0.001, HR = 0.198, 95% CI: 0.076–0.511) (**Figures 3A, B**). Furthermore, the 5-TAAbs panel remained to be a good predictive biomarker for patients treated with combination therapy (ORR: 43.8% vs. 13.7%, P = 0.004, PFS: 6.7 vs. 3.7m, *P* = 0.017, HR = 0.509, 95% CI: 0.303-0.857) (**Figures 3C, D**). Regarding to treatment-line, subgroup analysis indicated that this 5-TAAs panel worked better in patients who received subsequent-line ICIs-based treatment (ORR: 42.4% vs. 7.7%, *P* = 0.001, PFS: 6.2 vs. 3.0 m, *P* = 0.004, HR = 0.481, 95%CI: 0.295-0.785) (**Figures 3G, H**). Similar responses to first-line treatment (ORR: 46.7% vs. 35.7%, *P* = 0.345, PFS: NR vs. 10.48m, *P* = 0.146) between patients with positive or negative 5-TAAs might be mainly contributed by high proportion of combination therapy and small sample size (**Figures 3E, F**). In addition, for patients with EGFR mutation (n = 34), patients with 5-TAAbs positive has relatively longer median PFS and better responses than negative patients (6.2 vs. 3.7m, *P* = 0.196, HR = 0.527, 95% CI: 0.196–1.419, ORR: 20.0% vs. 9.1%, P = 0.572; DCR: 70.0% vs. 68.2%, *P* = 1.000) (**Supplement Figure**), though the differences did not reach the statistical significance. Univariate and multivariate analysis suggested that the 5-TAAbs positivity was an independent factor for PFS (**Table 2**).

**Table 2:** Univariate and multivariate analyses of clinical parameters of PFS in validation cohort.

Factors	Univariate analysis			Multivariate analysis		
	HR	95%CI	*P*	HR	95%CI	*P*
**Age,yr**						
≤63/>63	1.296	0.815-2.059	0.273			
**Gender**						
male/female	0.932	0.571-1.523	0.780			
**Smoking**						
heavy/no,light	0.944	0.561-1.589	0.828			
**ECOG**						
0-1/2-3	0.402	0.145-1.114	0.080	0.208	0.070-0.622	0.005
**Histology**						
non-squa/suq	0.958	0.562-1.632	0.875			
**Stage**						
IIIB-IIIC/IV	0.768	0.367-1.609	0.484			
**Metastasis**						
intrathoracic yes/no	1.017	0.636-1.627	0.943			
bone yes/no	1.552	0.880-2.738	0.129			
brain yes/no	1.238	0.709-2.162	0.454			
liver yes/no	1.791	0.942-3.403	0.075	1.843	0.953-3.564	0.069
others yes/no	0.930	0.561-1.541	0.777			
**PD-L1**						
1-49%/negative	0.593	0.245-1.436	0.247			
≥50%/negative	0.547	0.212-1.408	0.211			
**Genetype**						
EGFRm/WT	1.327	0.802-2.195	0.271			
**ICIs Lines**						
1/≥2	0.414	0.227-0.755	0.004	0.461	0.241-0.882	0.019
**Treatment**						
combination/mono	1.015	0.600-1.716	0.956			
**Autoantibodies**						
positive/negative	0.394	0.245-0.634	<0.001	0.413	0.253-0.675	<0.001

### Association Between 5-Tumor Associated Autoantibody Panel and CIP

For all patients included into the current study and followed up for more than 3 months (n = 122), CIP occurrence rate was 12.3%. The median onset time was 2.3 months (range 1.3–4.2 months). Comparing to negative patients, the CIP incidence in patients with 5-TAAbs positive was significantly higher (20.4% vs. 5.9%, *P* = 0.015) (**Figure 2C**). However, for all patients with CIP, the grades were similar between patients with positive and negative 5-TAAbs (**Figure 2D**). Significant higher risk of CIP occurrence was also observed in patients with positive 5-TAAbs (HR = 3.504, *P* = 0.032) (**Figure 2E**).

## Discussion

Predictive biomarkers of immunotherapy remain to be explored. Here, we identified a 5-TAAbs panel in a discovery cohort and subsequently confirmed its predictive value in a validation cohort. Like the recent study from Ohue and colleagues (15) that reported a strong correlation between serum antibodies (NY-ESO-1 and XAGE1) and clinical response to anti–PD-1 monotherapy for NSCLC, we have demonstrated the predictive value of 5-TAAbs panel for responses to both ICIs monotherapy and ICIs-based combination therapy for NSCLC in our study. While only 20%–25% of subjects in their study showed positive autoantibody results, our 5-TAAbs panel showed that about 50% of subjects were positive indicating a larger beneficial population would be covered. Besides, consistent with their results, we also did not observe a significant correlation between PD-L1 expression and TAAbs positivity. This may reflect two sets of predictive biomarkers with independent mechanistic pathways. One drawback of this study was that PD-L1 expression were detected by antibodies from different commercial companies, and other biomarkers such as TMB and tumor infiltration lymphocytes were not available for this study.

An explanation for the predictive role of autoantibody is that positivity of these TAAbs represented the high immunogenicity of corresponding TAAs, leading to antigen-specifical CD8+ T cells activation and checkpoint molecule–mediated strong immunosuppression, exactly as Ohue et al. isolated NY-ESO-1–specifical CD8+ T cell from peripheral blood of patient with NY-ESO-1 positive and speculated that antibody titers reflected cytotoxic activity levels of antigen-specific CD8+ T cell (15). Nonetheless, not all tumor antigens could elicit CD8+ T-cell responses. A previous study reported that mutant p53 peptides elicited CD4+ T cell and humoral, but not CD8+ T-cell responses (18). Tripartite motif-containing protein 21 (TRIM21) is an intracellular Fc receptor linking cytosolic antibody recognition to the ubiquitin proteasome system, which is also mainly involved in humoral immunity (19). Hence, another explanation for our finding is that pre-existing humoral immunity facilitated the anti-tumor activity of ICIs. TAAbs are produced by activated B lymphocytes stimulated by tumor autoantigens, which is an indication of active humoral immune response. Although for patients treated with ICIs, T cell-mediated immune reactive is considered as a prerequisite factor of anti-tumor activity, the role of humoral immunity has also been paid more attentions recently. Stankovic B and colleagues have shown that CD19+ B cells were the second most common immune cell type in NSCLC tumors (20). Suyama et al. reported a case of lung cancer patient with PR to nivolumab for more than seven months and immunohistochemical analysis of the metastatic lymph node biopsy specimen showed prominent accumulation of plasma cells and immunoglobulin G (21). These findings, together with our results suggested that pre-existing humoral immunity may be worth considering as a candidate therapeutic biomarker of ICIs in lung cancer patients.

Furthermore, unlike other studies focused on autoantibodies against to self-antigen, the current study was the first to report that autoantibodies against to TAAs was associated with CIP occurrence, which suggested that the preexisting active humoral response would also lead to excessive immune-attack and damage to self-tissue (12–14). However, as this was a retrospective study, periodic chest CT follow-up was difficult for every patient and thus certain percentages of patients with pneumonitis, especially with asymptomatic pneumonitis, may be neglected. Hence, our data need to be further validated in a large and perspective study. In addition, this 5-TAAbs panel had a trend in predicting clinical response of ICIs-based therapy among patients with EGFR mutations (25.0%) included in this study. As we all known, patients with EGFR mutations derives limited clinical benefits from ICIs-based treatment and even brought detrimental adverse reaction (22, 23). Up to now, the resistance mechanism is not clarified clearly and existing research attributed it to the low TMB and immune desert tumor microenvironment (24, 25). However, there are still some data suggest that some of these patients do respond to ICIs (26, 27). Although our results did not show statistical significance, which may attribute to the small sample size of this subgroup and possible different role for each antibody in such patients, we think that as a potential stratification biomarker, TAAbs, not limited to these 5 TAAbs in the present study, is worthy of further large-scale studies in EGFR mutant lung cancer patients.

In conclusion, we have identified a panel of 5-TAAbs and proved that this panel could predict clinical benefits of ICIs-based treatment in three centers from different regions of China. A diagnostic kit using this 5-TAAbs panel as a biomarker has been under development. The limitations of the present study were the small number of patients included, the short follow-up time after ICIs-based treatment and the lack of correlation analysis with other well-known biomarkers. Large clinical studies and further mechanistic research are needed to confirm the usefulness and rationality of the 5-TAAbs panel as a predictive biomarker for responses and toxicity to ICIs-base treatment.

## Data Availability Statement

The raw data supporting the conclusions of this article will be made available by the authors, without undue reservation.

## Ethics Statement 

The studies involving human participants were reviewed and approved by Shanghai Pulmonary Hospital. The patients/participants provided their written informed consent to participate in this study.

## Author Contributions

CS conceptualized the study and acquired the funding. JuZ, JiZ, QJ, and QC conducted the data curation and performed the formal analysis. JiZ, QJ, FZ, WZ, and XC acquired the clinical data and clinical samples. JuZ, JiZ, SR, CZ, and CS wrote, reviewed, and/or revised the manuscript. All authors contributed to the article and approved the submitted version.

## Funding

This study was supported by the National Natural Science Foundation of China (grant number: 81874036) and Science and Technology Commission of Shanghai Municipality (19411971100).

## Conflict of Interest

The authors declare that the research was conducted in the absence of any commercial or financial relationships that could be construed as a potential conflict of interest.

## References

[1] Arbour KC Riely GJ. Systemic Therapy for Locally Advanced and Metastatic Non-Small Cell Lung Cancer: A Review. JAMA (2019) 322(8):764–74. 10.1001/jama.2019.11058 31454018

[2] Garon EB Hellmann MD Rizvi NA Carcereny E Leighl NB Ahn MJ. Five-Year Overall Survival for Patients With Advanced Non-Small-Cell Lung Cancer Treated With Pembrolizumab: Results From the Phase I KEYNOTE-001 Study. J Clin Oncol (2019) 37(28):2518–27. 10.1200/JCO.19.00934 PMC6768611 31154919

[3] Gandhi L Rodríguez-Abreu D Gadgeel S Esteban E Felip E De Angelis F. Pembrolizumab plus Chemotherapy in Metastatic Non-Small-Cell Lung Cancer. N Engl J Med (2018) 378(22):2078–92. 10.1056/NEJMoa1801005 29658856

[4] Paz-Ares L Luft A Vicente D Tafreshi A Gümüş M Mazières J. Pembrolizumab plus Chemotherapy for Squamous Non-Small-Cell Lung Cancer. N Engl J Med (2018) 379(21):2040–51. 10.1056/NEJMoa1810865 30280635

[5] West H McCleod M Hussein M Morabito A Rittmeyer A Conter HJ. Atezolizumab in combination with carboplatin plus nab-paclitaxel chemotherapy compared with chemotherapy alone as first-line treatment for metastatic non-squamous non-small-cell lung cancer (IMpower130): a multicentre, randomised, open-label, phase 3 trial. Lancet Oncol (2019) 20(7):924–37. 10.1016/S1470-2045(19)30167-6 31122901

[6] Socinski MA Jotte RM Cappuzzo F Orlandi F Stroyakovskiy D Nogami N. Atezolizumab for First-Line Treatment of Metastatic Nonsquamous NSCLC. N Engl J Med (2018) 378(24):2288–301. 10.1056/NEJMoa1716948 29863955

[7] Hellmann MD Paz-Ares L Bernabe Caro R Zurawski B Kim SW Carcereny Costa E. Nivolumab plus Ipilimumab in Advanced Non-Small-Cell Lung Cancer. N Engl J Med (2019) 381(21):2020–31. 10.1056/NEJMoa1910231 31562796

[8] Li Y Zhang Z Hu Y Yan X Song Q Wang G. Pretreatment Neutrophil-to-Lymphocyte Ratio (NLR) May Predict the Outcomes of Advanced Non-small-cell Lung Cancer (NSCLC) Patients Treated With Immune Checkpoint Inhibitors (ICIs). Front Oncol (2020) 10:654. 10.3389/fonc.2020.00654 32656072 PMC7324627

[9] Möller M Turzer S Schütte W Seliger B Riemann D. Blood Immune Cell Biomarkers in Patient With Lung Cancer Undergoing Treatment With Checkpoint Blockade. J Immunother (2020) 43(2):57–66. 10.1097/CJI.0000000000000297 31592989 PMC7012348

[10] Cho YH Choi MG Kim DH Choi YJ Kim SY Sung KJ. Natural Killer Cells as a Potential Biomarker for Predicting Immunotherapy Efficacy in Patients with Non-Small Cell Lung Cancer. Target Oncol (2020) 15(2):241–7. 10.1007/s11523-020-00712-2 32285316

[11] Zhang Z Li Y Yan X Song Q Wang G Hu Y. Pretreatment lactate dehydrogenase may predict outcome of advanced non small-cell lung cancer patients treated with immune checkpoint inhibitors: A meta-analysis. Cancer Med (2019) 8(4):1467–73. 10.1002/cam4.2024 PMC6488146 30848091

[12] Gowen MF Giles KM Simpson D Tchack J Zhou H Moran U. Baseline antibody profiles predict toxicity in melanoma patients treated with immune checkpoint inhibitors. J Transl Med (2018) 16(1):82. 10.1186/s12967-018-1452-4 29606147 PMC5880088

[13] de Moel EC Rozeman EA Kapiteijn EH Verdegaal EME Grummels A Bakker JA. Autoantibody Development under Treatment with Immune-Checkpoint Inhibitors. Cancer Immunol Res (2019) 7(1):6–11. 10.1158/2326-6066.CIR-18-0245 30425107

[14] Toi Y Sugawara S Sugisaka J Ono H Kawashima Y Aiba T. Profiling Preexisting Antibodies in Patients Treated With Anti-PD-1 Therapy for Advanced Non-Small Cell Lung Cancer. JAMA Oncol (2019) 5(3):376–83. 10.1001/jamaoncol.2018.5860 PMC6439838 30589930

[15] Ohue Y Kurose K Karasaki T Isobe M Yamaoka T Futami J. Serum Antibody Against NY-ESO-1 and XAGE1 Antigens Potentially Predicts Clinical Responses to Anti-Programmed Cell Death-1 Therapy in NSCLC. J Thorac Oncol (2019) 14(12):2071–83. 10.1016/j.jtho.2019.08.008 31449889

[16] Tan Q Wang D Yang J Xing P Yang S Li Y. Autoantibody profiling identifies predictive biomarkers of response to anti-PD1 therapy in cancer patients. Theranostics (2020) 10(14):6399–410. 10.7150/thno.45816 PMC7255026 32483460

[17] Ren S Zhang S Jiang T He Y Ma Z Cai H. Early detection of lung cancer by using an autoantibody panel in Chinese population. Oncoimmunology (2017) 7(2):e1384108. 10.1080/2162402X.2017.1384108 29308305 PMC5749655

[18] Tsuji T Gnjatic S. Split T-cell tolerance as a guide for the development of tumor antigen-specific immunotherapy. Oncoimmunology (2012) 1(3):405–7. 10.4161/onci.19310 PMC3382850 22737632

[19] Rhodes DA Isenberg DA. TRIM21 and the Function of Antibodies inside Cells. Trends Immunol (2017) 38(12):916–26. 10.1016/j.it.2017.07.005 28807517

[20] Stankovic B Bjørhovde HAK Skarshaug R Aamodt H Frafjord A Müller E. Immune Cell Composition in Human Non-small Cell Lung Cancer. Front Immunol (2019) 9:3101. 10.3389/fimmu.2018.03101 30774636 PMC6367276

[21] Suyama T Fukuda Y Soda H Ogawara D Iwasaki K Hara T. Successful treatment with nivolumab for lung cancer with low expression of PD-L1 and prominent tumor-infiltrating B cells and immunoglobulin G. Thorac Cancer (2018) 9(6):750–3. 10.1111/1759-7714.12644 PMC5983224 29667757

[22] Oxnard GR Yang JC Yu H Kim SW Saka H Horn L. TATTON: a multi-arm, phase Ib trial of osimertinib combined with selumetinib, savolitinib, or durvalumab in EGFR-mutant lung cancer. Ann Oncol (2020) 31(4):507–16. 10.1016/j.annonc.2020.01.013 32139298

[23] Lee CK Man J Lord S Links M Gebski V Mok T. Checkpoint Inhibitors in Metastatic EGFR-Mutated Non-Small Cell Lung Cancer-A Meta-Analysis. J Thorac Oncol (2017) 12(2):403–7. 10.1016/j.jtho.2016.10.007 27765535

[24] Santaniello A Napolitano F Servetto A De Placido P Silvestris N Bianco C. Tumour Microenvironment and Immune Evasion in EGFR Addicted NSCLC: Hurdles and Possibilities. Cancers (Basel) (2019) 11(10):1419. 10.3390/cancers11101419 PMC6826622 31554160

[25] Soo RA Lim SM Syn NL Teng R Soong R Mok TSK. Immune checkpoint inhibitors in epidermal growth factor receptor mutant non-small cell lung cancer: Current controversies and future directions. Lung Cancer (2018) 115:12–20. 10.1016/j.lungcan.2017.11.009 29290252

[26] Gettinger S Hellmann MD Chow LQM Borghaei H Antonia S Brahmer JR. Nivolumab Plus Erlotinib in Patients With EGFR-Mutant Advanced NSCLC. J Thorac Oncol (2018) 13(9):1363–72. 10.1016/j.jtho.2018.05.015 29802888

[27] Hastings K Yu HA Wei W Sanchez-Vega F DeVeaux M Choi J. EGFR mutation subtypes and response to immune checkpoint blockade treatment in non-small-cell lung cancer. Ann Oncol (2019) 30(8):1311–20. 10.1093/annonc/mdz141 PMC6683857 31086949

