# Supplementary material for: Peripheral Blood Autoantibodies Against to Tumor-Associated Antigen Predict Clinical Outcome to Immune Checkpoint Inhibitor-Based Treatment in Advanced Non-Small Cell Lung Cancer
Source: Front Oncol. 2021 Mar 16;11:625578. doi: 10.3389/fonc.2021.625578 (PMC8010683; doi:10.3389/fonc.2021.625578)
Supplement: Supplementary file 1 [file DataSheet_1.pdf]

Supplementary Material

1 Supplementary Table

BRAF (P15056)	ZIC2 (O95409)	AKAP4 (Q5JQC9)	Cyclin D (P24385)	KOC (O00425)
IMP2 (Q9Y6M1)	P53 (P04637)	Annexin1 (P04083)	HSP105 (Q92598)	NY-ESO-1 (P78358)
KRT8 (P05787)	GAGE7 (O76087)	FEZF1 (A0PJY2)	IMP1 (Q9NZI8)	KK-LC-1 (Q5H943)
LY6K (Q17RY6)	CAGE (Q86TM3)	ENO1 (P06733)	MDM2 (Q00987)	
SOX2 (P48431)	MAGE-A4 (P43358)	BRCA1 (P38398)	TLK1 (Q9UKI8)	
MAGE-A3 (P43357)	XAGE (Q9HD64)	BRCA2 (P51587)	Claudin 2 (P57739)	
MAGE-A1 (P43355)	LAMR (P08865)	BRDT (Q58F21)	ETHE1 (O95571)	
PRAME (P78395)	Lengsin (Q5TDP6)	MYC (P01106)	P16 (P42771)	
TEX264 (Q9Y6I9)	SP17 (Q15506)	DKK1 (O94907)	CT46 (Q86X24)	
TTC14 (Q96N46)	Trim21 (P19474)	Cyclin B1 (P14635)	NY-ESO-2 (O75638)	

*these antigens showed high sensitivity (>5%) and specificity (93.6%) in NSCLC patients compared to healthy control subjects.

1.1 Supplementary Figure

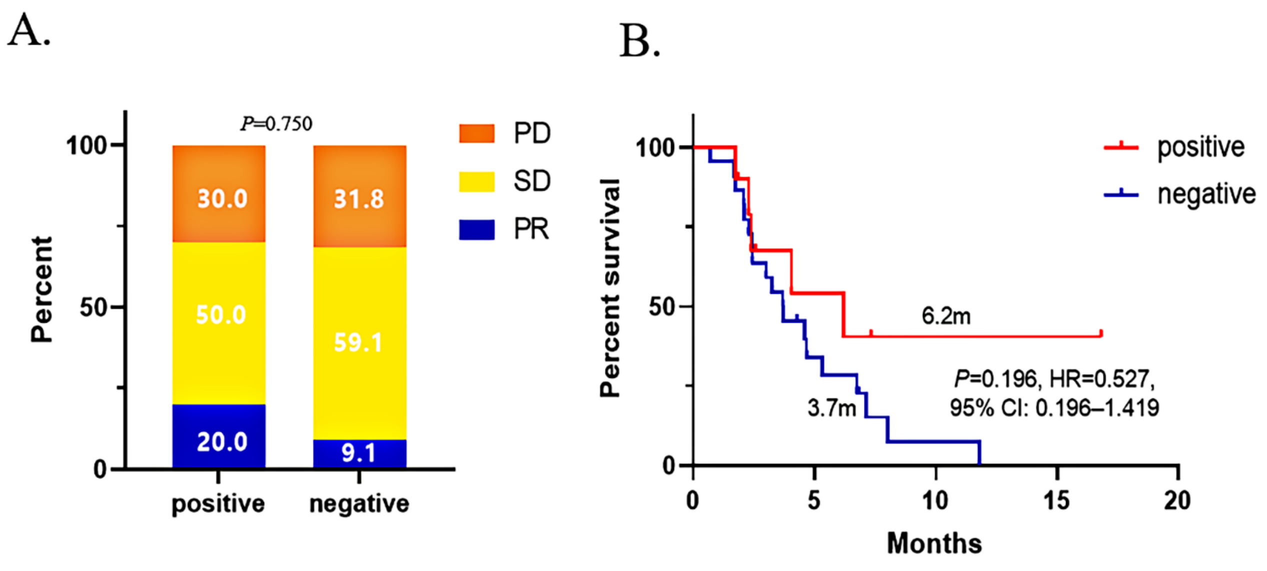

Supplementary Figure. ORR and PFS comparison of patients with 5-AABs positive or negative in subset of patients with EGFR mutation.
